# Galectin-3 Is a Crucial Immunological Disease Marker in Patients with Fungal Keratitis

**DOI:** 10.1155/2022/1380560

**Published:** 2022-07-08

**Authors:** Yichen Xiao, Jiahui Yang, Zhenyuan Fu, Dalian He, Naiyang Li, Jin Yuan

**Affiliations:** ^1^State Key Laboratory of Ophthalmology, Zhongshan Ophthalmic Center, Sun Yat-Sen University, Guangdong Provincial Key Laboratory of Ophthalmology and Visual Science, Guangzhou 510060, China; ^2^Eye Center, Zhongshan Hospital of Sun Yat-Sen University, Zhongshan, Guangdong, China

## Abstract

Fungal keratitis, one of the most common infectious eye diseases in China, often results in a poor prognosis due to a delayed diagnosis and the insufficiency of effective therapy. There is an urgent need to identify specific biomarkers for the disease. In this study, we screened out tear proteins in patients with fungal keratitis by microsphere-based immunoassay analysis. Levels of cytokine expression were determined in both human corneal epithelial cell models in vitro and the corneas of patients by western blot, quantitative polymerase chain reaction (qPCR), and immunofluorescence analysis. Neutrophil activation was examined by flow cytometry analysis. The relationship between the cytokine expression and neutrophils was evaluated by immunofluorescence costaining and correlation analysis. These results demonstrated that the galectin-3 expression level was increased in both cell model and patient samples at the early and late stages of fungal keratitis. The neutrophils were significantly activated during the disease course of fungal keratitis. Meanwhile, colocalization and a positive correlation between galectin-3 and neutrophils were observed, suggesting that galectin-3 may play a crucial role in the recruitment of neutrophils and immune regulation of fungal keratitis. In conclusion, galectin-3 could be a key disease marker implying a beneficial immune response in the pathogenesis of fungal keratitis, which might be a target of therapeutic strategy in the future.

## 1. Introduction

Fungal keratitis (FK), also known as mycotic keratitis, is a severe corneal infection that often results in permanent blindness and vision loss. It is estimated that there are 1051787 new FK cases each year worldwide, with the highest incidence rates in Asia and Africa. The incidence of FK in China is more common than in other regions [1]. The immune responses of fungal keratitis include both anti-infection responses and immune disorders after pathogen invasion. Our previous study demonstrated that triggering receptors expressed on myeloid cells-1 (TREM-1) and dendritic cell-associated C-type lectin-1 (Dectin-1), the vital pattern recognition receptors (PRRs) in the innate immune response, initiated the whole anti-infection process, positively participated in the pathogenesis of ocular fungal infection, and provided the foundation of the adaptive immune response [2–4]. However, the mechanism of innate immune response in FK remains unveiled to a great extent [5].

On the ocular surface, where the battlefield of FK is, the inherent corneal epithelial cells, together with macrophages, neutrophils, and T effective immune cells recruited from the systemic immune system, fight against invading pathogens [6]. Galectin-3 is a soluble mammalian lectin that positively modulates the immune response against pathogenic microorganisms, including bacteria and fungi [7, 8]. It is implicated that galectin-3 can activate chitin-responsive pattern recognition receptors and regulate signal transduction, thereby playing a vital role in initiating an anti-infection inflammation cascade in the host immune system [9, 10]. The fungal infection leads to an inflammatory cascade of signal transduction events, including infiltration of inflammatory cells like neutrophils and inflammatory cytokines necessary to initiate adaptive immunity and to kill and clear the invading fungi [11]. The activation and recruitment of neutrophils run throughout the whole antifungal defense, and neutrophils are the main force fighting against fungal invasion as well as responders to proinflammatory cytokines [2]. Since galectin-3 functions as a regulatory molecule in acute and chronic inflammation, it is well-accepted that it acts as an amplifier of the inflammatory cascade with its accelerative role in neutrophil chemotaxis. However, its role in fungal keratitis and its potential synergetic mechanism with neutrophils remain unknown [7, 12, 13]. We deemed that further studies on the innate immune system should be performed to discover more potential immunological targets to unveil and enrich the pathogenesis of fungal keratitis.

This study was aimed at identifying potential disease markers of FK by evaluating the levels of tear cytokines, validating the candidate biomarker in both fungal infection cell model and tissue biospecimens obtained from FK patients, and confirming its role in the blood sample and focal corneal tissue. Our findings provide a new biomarker for the prediction of the development of FK and the regulation of immune responses in FK. The current study will also shed light on the development of a novel therapeutic strategy against FK.

## 2. Materials and Methods

### 2.1. Patient and Tissue Specimens

The tissue samples were collected from patients who had clinically diagnosed fungal keratitis by corneal scraping culture and received corneal transplantation from May 2020 to May 2021 at the Zhongshan Ophthalmic Center. This study was approved by the Zhongshan Ophthalmic Center Medical Science Research Ethics Committee (protocol number: 2020KYPJ115). All participants in this study provided written consent. The infected corneal tissues were collected from the corneal transplantation surgery. Tears and peripheral blood specimens were collected from the Department of Cornea and Ocular Surface Disease at Zhongshan Ophthalmic Center. Normal donor corneas were obtained from the Guangdong Eye Bank. Samples were quickly stored in a cryogenic refrigerator at -80°C. All medical records, clinical signs, anterior segment photography, and follow-up data were complete. The patients were divided into two groups based on the severity of the ulcer: 9 cases with the partially infiltrated cornea and no anterior chamber empyema in the early stage group and 22 cases with total corneal infiltration or anterior chamber empyema in the late stage group. The severity of fungal keratitis was graded on a scale ranging from 0 to 12 according to a scoring system developed by Zhou et al. [14]. Four clinical scores were calculated by aspects: diameter of corneal ulcer (0-3), infiltrating depth (0-3), corneal edema (0-3), and the response of the anterior chamber (0-3).

In this study, a patient with clinical symptoms of suppurative keratitis was considered to be a case of FK if the patient met one or more than one of the following criteria at the initial visit: (1) grown fungal organism from a corneal scrape or biopsy sample in one or more culture media, or featured fungal elements shown in light microscopy of corneal scrape samples, and (2) fungal elements identified by in vivo confocal microscopy (IVCM). Subjects were excluded if they had (1) coexistence of bacteria organism determined by culture results, (2) presence of corneal endophthalmitis and specific perforation on the day that cultures were taken, (3) presence of immune disorders, autoimmune diseases, allergic diseases (such as Mooren's corneal ulcer), and (4) not willing to participate, pregnancy, or breastfeeding.

### 2.2. Microsphere-Based Immunoassay Analysis

To measure the minimal concentrations of tear cytokines, we performed a microsphere-based immunoassay analysis with a multifunctional liquid suspension chip system from Luminex to test different cytokine levels simultaneously. The patients' tear samples were collected in a quiet room at 17 : 00 each day. With no epithelial anesthetics, sterile capillary tubes were used to collect fluid tear samples from the edge of the lower eyelid under a slit lamp. 2 *μ*L fluid tear samples of involved patients were collected from each eye and immediately stored in a cryogenic refrigerator at -80°C to avoid repeated freezing and thawing. We used the Luminex 200™ system (Luminex, Austin, TX, USA) to detect cytokine levels in tear samples, including galectin-3, IL-1*β*, IL-18, and TNF-*α*, and three replicate analyses were performed. Luminex 200 IS V2.1 software was applied to obtain the concentration value from the mean fluorescence intensity (MFI). After a standard curve was generated concerning cytokine gradient concentration, the concentrations of the cytokines listed above in the tear samples were calculated according to the standard curve.

### 2.3. In Vitro Cell Model

The corneal epithelial cell line HCEC (human corneal epithelial cell) used in this study was purchased from Cyto-Biotech. HCECs were seeded into flasks and put into a humidified incubator with 5% carbon dioxide (CO2) at 37°C. The cell culture medium is made up of Dulbecco's modified Eagle's medium (DMEM)/F12 cell culture medium (Gibco, Grand Island) containing 1% sodium pyruvate, 10% fetal bovine serum, 1% human epidermal growth factor, 1% streptomycin, and penicillin that were added to the culture medium. Confluent cells were subcultured every 2–3 days by trypsinization with a 0.5% trypsin/EDTA solution. An in vitro model of fungal keratitis was established by treating corneal epithelial cells with zymosan, a yeast polysaccharide (100 mg/mL) for 24 h [2, 15, 16]. Total proteins or RNA was extracted from cells after treatment for further examination.

### 2.4. Immunofluorescence Staining

Immunofluorescence staining was performed in both human corneal epithelial cell line and corneal tissues from patients with fungal keratitis to detect the expression of cytokeratin 12 (CK12), CD11b, and galectin-3. After being stimulated by yeast polysaccharide (100 mg/mL) for 24 h, cell cultures were cooled at 4°C, then washed with PBS, and fixed overnight with methanol at −20°C. After treatment with acetone for 1 minute, the cells were dried in the open air. The cells were rehydrated in IMF buffer (0.1% Triton X-100, 0.15 mol/L NaCl, 5 mmol/L EDTA, 20 mmol/L HEPES, and 0.02% NaN3) and incubated at 4°C overnight with a mixture of mouse anti-CK-12 (rabbit, Bioss, bs-4625R; 1 : 50) or mouse anti-CD11b (rabbit, Bioss, bs-1014R; 1 : 50) and rabbit anti-galectin-3 (mouse, ThermoFisher, 395100; 1 : 25) antibodies. The cells were then washed and incubated for 1 h at room temperature with secondary antibodies Dy-Light 594 goat anti-mouse IgG (Vector Laboratories, DI-1094; 1 : 200) and streptavidin-FITC (Southern Biotech, 7100-02; 1 : 50) in HEPES solution for more than 1 hour at room temperature, followed by rinsing with PBS. Nuclei were freshly stained with DAPI (Millipore Sigma), and tissue sections were covered with a set of coverslips. Images were acquired with a BZ-X700 all-in-one fluorescence microscope (Keyence).

The corneal tissues of patients with fungal keratitis were prepared by frozen sectioning techniques. After washing with distilled water, tissue sections were blocked with a 3% BSA blocking solution, according to the reagent instructions (Abcam). Sections were incubated with primary antibodies for a mixture of CK12 (rabbit, Bioss, bs-4625R; 1 : 100) or CD11b (rabbit, Bioss, bs-1014R; 1 : 100) and galectin-3 (mouse, ThermoFisher, 395100; 1 : 200) in HEPES solution and then supplemented with 2.5% horse serum overnight at 4°C. After careful washing, tissue sections were incubated with the secondary antibody (1 : 200). The subsequent operation is the same as the cell slide treatment.

### 2.5. Flow Cytometry Analysis

Flow cytometry was performed to determine the proportion of neutrophils in peripheral blood at different stages of fungal keratitis. Cell blood samples were washed twice at 1000 rpm for 5 min at 4°C in PBS. The cell suspension was resuspended in Thermo lysing buffer for 60s to eliminate red blood cells out of cell suspension, and it was centrifuged again at 1000 rpm for 5 min at 4°C in PBS and then stained with antibodies in the dark. Fc receptors were blocked for 15 min at RT with anti-mouse or anti-human CD16/32 antibody (e-Bioscience 16-0161-81) followed by incubation with FITC tagged anti-CD11b antibody and PE tagged anti-CD66b antibody (Bio-legend, 127613, Bio-legend, 561650, 0.5 *μ*g added to 1 × 10^6^ cell suspension in 100 *μ*L). After two washes in FACS buffer (1% FBS in PBS), the cells were fixed in 0.5% PFA, then washed, resuspended in flow cytometry staining buffer and analyzed by flow cytometry on a FACs Aria (BD Biosciences). Data were analyzed using BD FACSDiva software.

### 2.6. Western Blot Analysis

To measure the protein level of galectin-3 in both corneal epithelial cells and tissues from patients with fungal keratitis, cellular and tissue lysates were prepared by lysing samples in ice-cold RIPA buffer (25 mM Tris-HCl pH 7.6, 0.15 M NaCl, 1% IGEPAL CA-630, 1% sodium deoxycholate, 0.1% SDS) containing 1%PMSF. Protein extraction was precisely performed using a Pierce BCA Protein Assay Kit (Tiangen, Beijing, China). For each sample, 40 *μ*g of protein was loaded into each well. Sodium dodecyl sulfate-polyacrylamide gel electrophoresis (SDS-PAGE) was performed with 12% gels to separate proteins. Proteins were transferred to a polyvinylidene difluoride (PVDF) membrane (Invitrogen, USA), which was then blocked for 2 h with 5% bovine serum albumin (BSA). The membrane was then incubated with rat anti-mouse galectin-3 mAbs (Abcam, UK) overnight at 4°C. On the next day, the membrane was washed and incubated with anti-rat or peroxidase-conjugated secondary antibodies (Abcam, UK) for 1 h. Immuno-reactive bands were visualized in an Odyssey Infrared Imaging System (LI-COR Biosciences, Frederick, MD) according to the supplier's instructions. GAPDH was used as the loading control. The integrated density of bands in the obtained results was further quantitatively analyzed by ImageJ.

### 2.7. Quantitative RT-PCR

To examine galectin-3 mRNA expression in fungal keratitis patients, we used a Qiagen RNA extraction kit to extract total RNA from the corneas of fungal keratitis patients, according to the instructions. RNA reverse transcription was performed with the TaKaRa Reverse Transcription Kit on a PCR machine. Transcripts were quantified with SYBR Green qPCR reagents on an IQ Thermocycler (Bio-Rad) or ABI Quant Studio Design v1.3. PCR primers were purchased from Invitrogen, and their sequences are shown below:

- Galectin-3: forward: 5′-GCTTATCCTGGCCCAACTGC-3′, reverse: 5′-CCCCGCTGGACCACTGACGG-3′
- GAPDH: forward: 5′-TGTGGGCATCAATGGATTTGG-3′, reverse: 5′-ACACCATGTATTCCGGGTCAAT-3′

### 2.8. Statistical Methods

Normally distributed data (such as age and disease course) were analyzed by mean ± SD and an independent *t*-test. Qualitative data, including gender and the number of the affected eyes, were compared using the chi-square test. Paired *t*-tests were utilized to analyze the differences in the cytokine levels and cell proportion in normal vs. affected eyes and the early vs. late stage of fungal keratitis. Spearman's tests were used for correlation analysis between the levels of galectin-3 in tears and neutrophils in peripheral blood of patients with fungal keratitis. Statistical analyses were performed using SPSS for Windows (version 22.0; International Business Machines Corporation). Graphs were generated by GraphPad Prism 7 for Windows (version 7.04; GraphPad Software). *P* values were considered significant when less than 0.05.

## 3. Results

### 3.1. Galectin-3 Significantly Expressed in Tears of Patients with Fungal Keratitis

**Figure 1:**
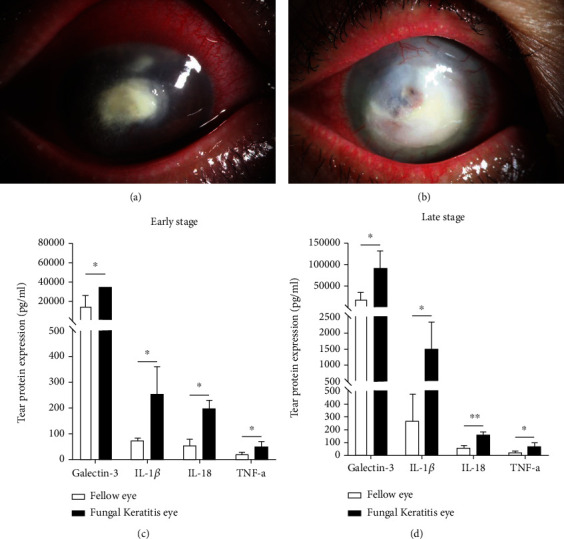
Morphology of human fungal keratitis in the early (a, *n* = 9) and late (b, *n* = 22) stages (tear protein levels were measured with more than 3 repeats). Samples of each patient were collected 3 times repetitively. Protein levels of galectin-3 and related inflammatory factors IL-1*β*, IL-18, and TNF-*α* in tears of patients with fungal keratitis. The expression of these factors was increased in both the early (c) and late stages (d) compared to the fellow eyes. ∗: *P* < 0.05; ∗∗: *P* < 0.01; ∗∗∗: *P* < 0.001; NS: no statistical difference.

Thirty-one fungal keratitis patients with a mean age of 61.30 ± 2.55 years (males 33.33%, females 67.67%) in the early stage and 62.05 ± 1.47 (males 40.91%, females 59.09%) in the late stage were recruited into the study. Right eye data and left eye data counted for 67.7% and 33.3%. The data of fellow eyes of disease patients without systemic immune disease were collected as the control group. Patients were divided into 2 groups, 9 patients in the early stage group and 22 patients in the late stage group.  Figure 1(a) showed the patients in the early stage had typical signs such as localized infiltration of inflammatory cells, fungal pseudopodia, and corneal conjunctival hyperemia. While in the late stage (Figure 1(b)), the infiltration was further aggravated to hypopyon, with ulcers involving the whole anterior chamber. The average disease score of the early stage group is 9.90 ± 1.05, while of the late stage is 37.41 ± 4.04 (*P* < 0.001). The peak incidence of severe symptoms and signs is significantly higher in late stage patients, resulting in a higher average clinical score.

To further investigate the possible key disease markers in the pathogenesis of fungal keratitis, the levels of tear cytokines, which were found to be different between the early and late stages in fungal keratitis patients, were tested with microsphere-based immunoassay analysis. Through the analysis of tear cytokines, four cytokines were found to have statistically significant differences, including IL-18, IL-1*β*, galectin-3, and TNF-*α*. The expression of these inflammatory factors increased in the early and late stages compared with the fellow eyes. The expression levels of four factors in the tears of patients with fungal keratitis in the early stage are as follows: galectin-3, 36107.60 ± 6116.71 pg/mL (*P* < 0.05); IL-1*β*, 242.76 ± 105.73 pg/mL (*P* < 0.05); IL-18, 194.57 ± 29.45 pg/mL (*P* < 0.05); and TNF-*α*, 49.91 ± 17.57 pg/mL (*P* < 0.05) (Figure 1(c)) while galectin-3, 87507.08 ± 31474.39 pg/mL (*P* < 0.05); IL-1*β*, 1526.30 ± 766.98 pg/mL (*P* < 0.05); IL-18, 148.11 ± 28.99 pg/mL (*P* < 0.01); and TNF-*α*, 70.29 ± 27.73 pg/mL (*P* < 0.05) in the late stage (Figure 1(d)), respectively. The results also showed that the expression levels of IL-1*β*, galectin-3, IL-18, and TNF-*α* were higher than the healthy control. Both in the early and the late stages, galectin-3 increased most significantly among these proinflammatory cytokines. It is noteworthy that the expression level of galectin-3 was the highest in both early and late stages among detected cytokines, suggesting that it may play an important role in fungal keratitis.

### 3.2. Galectin-3 Expressed Highly in Both Cell Model In Vitro of Fungal Keratitis and the Corneas of Patients with Fungal Keratitis

**Figure 2:**
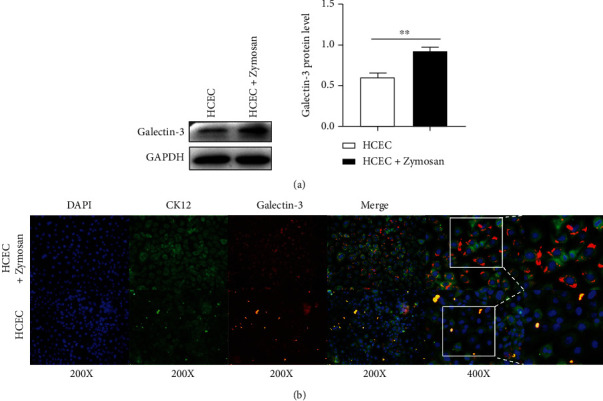
The expression of galectin-3 protein determined by western blots and statistical difference in galectin-3 expression of WB results of corneal epithelial cells increased in the presence or absence of zymosan (a). Colocalization of CK12 and galectin-3 expression in corneal epithelial cells was determined by immunofluorescence analysis (b). There were more than 3 repetitive samples of each group. ∗: *P* < 0.05; ∗∗: *P* < 0.01; ∗∗∗: *P* < 0.001; NS: no statistical difference.

To further investigate the role of galectin-3 in fungal keratitis, we determined the expression of galectin-3 in cell model in vitro of fungal keratitis and in corneal tissues collected from patients with fungal keratitis. In the cell model, human corneal epithelial cells were treated with zymosan (a yeast polysaccharide) for 24 h. The western blot data showed that the expression of galectin-3 in zymosan-treated cells was significantly higher than that in the control group, which was proved by the relativity analysis of the integrated density (HCEC vs. HCEC+zymosan: 0.63 ± 0.05 vs. 0.87 ± 0.05, *P* < 0.01) (Figures 2(a)). Immunofluorescence results showed that galectin-3 signals (red fluorescence) were primarily localized on the plasma membrane of the corneal epithelial cells labeled CK12 (green fluorescence), and the galectin-3 expression was significantly increased after the zymosan treatment (Figure 2(b)).

**Figure 3:**
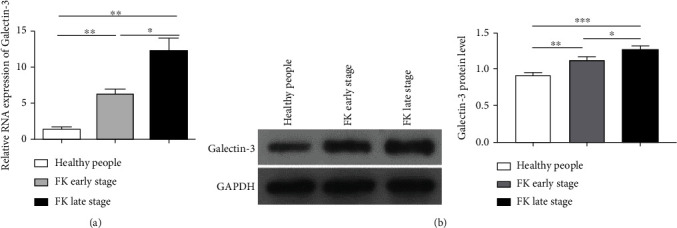
The expression of galectin-3 at the mRNA and protein level in the cornea of patients with fungal keratitis in early and late stages increased significantly, which was determined by qPCR (a) and western blot (b), respectively. There were more than 3 repetitive samples in each group. ∗: *P* < 0.05; ∗∗: *P* < 0.01; ∗∗∗: *P* < 0.001; NS: no statistical difference.

Next, we analyzed galectin-3 expression in corneal tissues collected from corneal transplantation surgery of patients with fungal keratitis. Real-time PCR analysis (Figure 3(a)) showed that the expression of galectin-3 RNA was significantly upregulated in corneas infected by fungus, with a 4-fold increase in the early stage (6.20 ± 1.31) (*P* < 0.01) and a 10-fold increase in the late stage (12.00 ± 3.61) (*P* < 0.01) compared to normal controls (1.03 ± 0.25). Western blot also showed that the levels of integrated density of galectin-3 expression increased gradually during the course of disease (healthy people vs. FK early stage: 0.92 ± 0.04 vs. 1.12 ± 0.05, *P* < 0.01; healthy people vs. FK late stage: 0.92 ± 0.04 vs. 1.27 ± 0.05, *P* < 0.001; FK early stage vs. FK late stage: 1.12 ± 0.05 vs. 1.27 ± 0.05, *P* < 0.05) (Figure 3(b)), consistent with the PCR results. Together, the results from both in vitro cell-based and human corneal tissue-based studies indicate that galectin-3 is significantly upregulated in fungal keratitis, and the expression is positively correlated with the disease progression, suggesting that galectin-3 may play an important role in fungal keratitis.

### 3.3. Galectin-3 Recruited Neutrophils from the Peripheral Blood in Fungal Keratitis Patients

**Figure 4:**
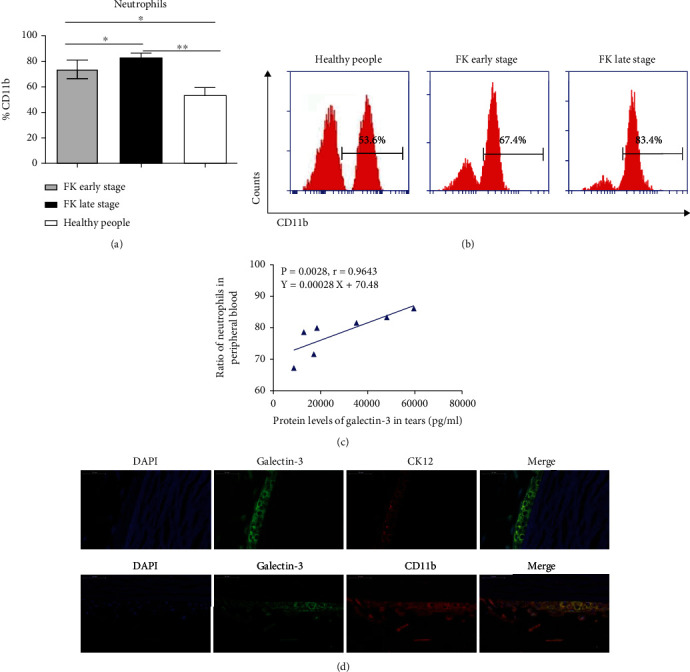
Analysis of neutrophil activation (CD11b) in peripheral blood at both the early and late stages of fungal keratitis and its correlation with the expression of galectin-3. The proportion of neutrophil activation increased gradually with the prolongation of the disease course (a and b). Correlation analysis (c) as well as immunofluorescence costaining (d) of galectin-3 (green fluorescence) and corneal epithelial cells (CK12, red fluorescence) or neutrophil (CD11b, red fluorescence) indicated that galectin-3 and neutrophil recruitment were highly correlated. There were more than 3 repetitive samples in each group. ∗: *P* < 0.05; ∗∗: *P* < 0.01; ∗∗∗: *P* < 0.001; NS: no statistical difference.

To explore the immune mechanisms underlying galectin-3 in the regulation of fungal keratitis, we first analyzed neutrophils, which play a significant role in the innate immunity of keratitis, at different stages of the disease. Flow cytometry was used to analyze the proportion of neutrophils in peripheral blood samples of fungal keratitis patients. The results showed that the cell frequency of neutrophils (CD66b, CD11b) significantly increased with the progression of the disease. The proportions of CD11b positive neutrophils in the CD66b positive group were 73.63 ± 7.19% at the early stage and 82.73 ± 3.75% at the late stage, both of which were significantly higher than that of the normal controls (53.67 ± 5.90%) (*P* < 0.05) (Figures 4(a)–4(c)). The results indicated that neutrophils play a vital role in the pathogenesis of fungal keratitis.

Next, we analyzed the potential correlation between the expression of galectin-3 and neutrophil activation in peripheral blood samples of patients with fungal keratitis. The results showed that galectin-3 expression was positively correlated with the proportion of neutrophil frequency in peripheral blood samples (*Y* = 0.00028x + 70.48, *R* = 0.9643, *P* < 0.01) (Figure 4(d)). To confirm this, frozen corneal tissue sections were collected from patients after corneal transplantation. The relationship between the expression of galectin-3, human corneal epithelium-labeled protein CK12, and neutrophil-labeled protein CD11b was determined by immunofluorescence costaining. We observed that galectin-3 (green fluorescence) is mainly expressed in the corneal stromal layer and the corneal epithelium layer (CK12, red fluorescence), and CD11b (red fluorescence) is also expressed in both two layers (Figure 4(d)). Galectin-3 and CK12 were co-expressed in the corneal epithelium, while galectin-3 and CD11b were aggregated near the corneal ulcer. Elevated expression of galectin-3 and its localization to the corneal epithelium and stroma suggested that galectin-3 may play an important immune regulatory function by recruiting and activating neutrophils in fungal keratitis.

## 4. Discussion

Fungal keratitis is an ocular infection disease. Its pathogenic mechanism mainly emphasizes chronic inflammation and immune dysregulation [17]. Our results demonstrate that galectin-3, an essential immunological marker, participated in the immune responses and increased obviously in different stages of fungal keratitis. Moreover, neutrophils were intensely activated in the course of fungal keratitis, and the positive correlation and colocation of galectin-3 and neutrophils suggested that galectin-3 may play an immune regulatory role by recruiting neutrophils in the early and late stages of fungal keratitis.

The role of galectin-3 in fungal keratitis has not been studied previously. Our finding of a significant increase of galectin-3 in tear samples of patients with fungal keratitis suggests that galectin-3 might be an indicator of the initiation of immune reactions in human fungal keratitis. The expression of galectin-3 in the HCEC cell model infected by zymosan, the specific antigen of fungi, suggests that human corneal epithelial cells secrete galectin-3 after recognizing the fungi invasion. It has been reported that the recruitment of neutrophils to the site of infection is critical for the clearance of the invading fungi, although other factors governing the host response are responsible for sensing the fungal invasion [18]. Galectin-3 has been reported as a markedly upregulated factor in mice fungi pulmonary infection. It possibly regulates fungal clearance mainly by promoting neutrophil activation migration in the lung fungal infection mice model [19, 20]. This research agrees with our data on the correlation between neutrophils and galectin-3 in FK. In the innate immune system responses to fungal infection, both the inherent corneal epithelial cells and neutrophils are recruited from the systemic immune system to fight together against invading pathogens [5]. Increased expression of galectin-3 in both tear samples and the cell model of FK supports the hypothesis that galectin-3 participates in the activation of inherent HCEC cells.

Speaking of bacterial keratitis and viral keratitis, there is no specific research on galectin-3 in animal models of the viral keratitis or the bacterial keratitis, but in the human epithelial cell models, galectin-3 is produced and expressed [21]. Galectin-3 contributes to maintaining innate barrier function and supports its physical defense [22]. Galectin-3 reduced the combined efficiency of virus and bacterial infections, such as HSV-1 and P. aeruginosa [23]. Galectin-3 participates in the mechanism of viral infections, such as human immunodeficiency virus (HIV) and influenza virus infections in viral binding, replication, budding, and transmission [24]. Some studies have revealed a direct antimicrobial effect of galectin-3 on the bacteria for galectin-3-binding caused bacterial cell death and bacterial growth suppression [25–27]. A protective role of extracellular galectin-3 against S. pneumoniae infection was proved in a bacterial infection model. For the reason that galectin-3 is involved in key immune cell subtypes in inflammatory responses, including neutrophil activation and adhesion [28], such as chemoattraction of monocytes and macrophages [29] and opsonization of apoptotic neutrophils [30], galectin-3 is vital in the immunological mechanism of inflammatory diseases.

On the other hand, our data on FK patient peripheral blood samples confirmed that the increased tendency of neutrophils is significantly elevated compared to healthy patients both at the early and late stages. The co-location expression results of galectin-3 and the typical markers of neutrophils (CD11b), and HCECs (CK12) reveal the co-resistance of galectin-3 and neutrophils in fungal keratitis lesions. Even though the blood-eye barrier limits the frequent recruitment of immune cells, the certain correlation between galectin-3 and neutrophils implies that targeting galectin-3 could be a promising alternative for FK treatment during the different courses. It is reported that members of the galectin family are able to crosslink cell-surface glycoconjugates with *β*-galactoside, resulting in the regulation of signaling, adhesion, and cell survival of immune cells such as neutrophils [7]. A specific alternative spliced form of galectin-3 has been reported to contain a predicted transmembrane-spanning domain. The neutrophils play a key role in immune responses caused by fungi infection. For the well-recognized key pathogenic role of neutrophils in FK, a novel role in host defense against fungal infections of ocular disease is found. The elevated galectin-3 expression might be an indicator of the inflammatory response against fungal infection. Thus, a decreased galectin-3 expression might be a “red light” signal of immunosuppressive drug abuse. Galectin-3 can serve as a sensitive biomarker for clinicians to make adjustments for better drug therapy [31, 32]. Besides, the increased levels of proinflammatory cytokines (IL-18, IL-1*β*, and TNF-*α*) in tear samples of FK patients, suggesting NLRP3 inflammasome activation, also contributes orchestration of neutrophil recruitment depending upon TLR-induced inflammatory cascade [33, 34].

It is controversial that it is whether the yeasts or hyphae are the certain forms of the pathogen to cause corneal infections; it is reported that yeasts are likely to activate immune response [35], while hyphae can activate the immune response and cause corneal stroma melting and corneal perforation at the same time; the expression levels of inflammatory factors could be affected by the forms of fungi [35]. With accumulating evidence, hyphae form is an essential pathogen agent in fungal infectious diseases. In a recent study, for gut fungal infection, the transition between yeast and invasive hyphae is crucial to fungal toxicity. The master regulator of hyphae-specific markers regulated the disease-causing potential of the pathogen. The formation of yeast-to-hyphae is essential for its pathogenesis [36]. It is also reported that fungi tended to provoke inflammatory responses only upon morphological transformation to their hyphal appearance [37]. Moreover, in skin lesions of fungal infection, the presence of hyphae is correlated with the intense production of proinflammatory cytokines [38]. We assumed that yeasts may exist in the early stage of disease and scar healing stage, but fungi thrive and grow robustly in the stage of disease progression, so the forms of fungi in this stage would be mixed. It could be a factor of the reason why the levels of inflammatory factors in tears are different from the early stage to the late stage, which is the reason why the form of fungi in the progression stage is mixed (both yeasts and hyphae). It could be a factor to affect inflammatory factor expression in tear samples in the early stage and the late stage.

In this study, for microsphere-based immunoassay analysis protein examination in tear samples, 9 patients with fungal keratitis in the early stage and 22 patients with fungal keratitis in the late stages were recruited. However, more patients could be recruited in future studies. For the limited quantity and size of corneal tissues taken from corneal transplantation surgeries, the RNA and protein expression of key disease markers were examined, while the expression levels of other markers remained to be done. With more accumulated corneal samples of fungal keratitis patients, future research should be undertaken to explore more essential biomarkers using RNA-seq, CO-IP, LC-MS, and so on.

There is accumulating evidence that galectin-3 regulates TLR activation and PAMPs at the beginning of host defense and inflammatory cascades [32]. Innate immune responses, including antigen-presenting, cell survival, ROS production, macrophage phagocytosis, cytokine production, and the microbial killing neutrophil generation take place sequentially in the pathogenesis of fungal keratitis [39, 40]. Our finding indicates the interactions between neutrophils and galectin-3. It has not been reported that galectin-3 regulates neutrophil functions, including recruitment, killing, and response to immune-modulatory cytokines in the fungal keratitis model in vivo. We intend to explore this mechanism in detail in the near future.

## 5. Conclusion

Conclusively, our results demonstrated that galectin-3 could be a significant and promising disease marker in fungal keratitis, and the galectin-3 modulation correlated with neutrophils in fungal keratitis at the early and late stages. In other words, galectin-3 might be a promising target for fungal keratitis therapeutic strategy in the future, which will shed some light on fungal keratitis patients, who are first to care.

## Data Availability

The data and materials used to support the findings of this study are available from the corresponding author upon request.
